# Applying the Balanced Scorecard to Build Service Performance Measurements of Medical Institutions: An AHP-DEMATEL Approach

**DOI:** 10.3390/ijerph20021022

**Published:** 2023-01-06

**Authors:** Chieh-Yu Lin, Fu-Chiang Shih, Yi-Hui Ho

**Affiliations:** 1 Department of International Business, Chang Jung Christian University, Tainan 71101, Taiwan; 2 Ph.D. Program in Business and Operations Management, Chang Jung Christian University, Tainan 71101, Taiwan

**Keywords:** service performance, medical institutions, balanced scorecard (BSC), analytic hierarchy process (AHP), decision making and trial evaluation laboratory (DEMATEL)

## Abstract

The main purpose of this study is to explore the application of the balanced scorecard (BSC) to service performance measurements of medical institutions using the analytic hierarchy process (AHP) and decision making and trial evaluation laboratory (DEMATEL). According to the concept of BSC, a total of four evaluation dimensions and twenty-two indicators of medical service performance measurements were developed. To collect data, this study delivered expert questionnaires to medical-related professional supervisors, deans, and heads of medical institutions in Taiwan. By combining the AHP and DEMATEL, the priority and causality of service performance standards in medical institutions were obtained. The results of this study show that the customer dimension is the most important service performance measurement dimension for medical institutions. The seven key service performance measurement indicators that are most important for medical institutions, in order, are “complete and comfortable equipment”, “competitiveness of the medical profession”, “continuity of patient-to-hospital treatment”, “classification of medical profession according to customers (VIP system)”, “complete medical service”, “complete salary, remuneration, and policy”, and “medical incomes of institutions”. In terms of causality, provided the complete services of medical institutions are improved, the continuity of patient-to-hospital treatment, the competitiveness of the medical profession, and the medical incomes of institutions would be influenced.

## 1. Introduction

In the past few decades, with rapid changes in the operation environment of medical institutions, improving the quality of medical services and reducing the incidence rate of adverse medical events have been the most noteworthy issues in the field of medical services. In addition, due to the rapid aging of the population, the increasing cost of medical treatment for various senile diseases, as well as the extensive use of expensive medical services, the income and expenditures of medical institutions are in an unbalanced state. Therefore, the primary focus of health care officials and medical institution managers is to constantly control costs and expenditures and verify the potential benefits of performance indicators for medical service quality. With the establishment of a large number of measurement indicators, various difficulties and shortcomings have been pointed out in the management of medical institutions [1]. As medical institutions are required to achieve higher service efficiency and medical quality when providing medical services to patients, medical service providers in medical institutions are expected to work long hours, which leads to fatigue caused by overworking, thus reducing their work enthusiasm and satisfaction [2].

With continuous and long-term efforts, the utilization rate of equipment may be increased, which will have an impact on the physical and mental health of staff. The severity of patients’ diseases and the short cycle from hospital admission to discharge pose a severe challenge to the provision of safe and effective medical services [3], and the high frequency of medical–patient relationships will inevitably lead to higher medical risks, especially in small hospitals and clinics. It is difficult for a medical institution to take full charge of medical service issues and solve all types of medical service management problems. Moreover, it is difficult to evaluate clinical and medical services according to performance when promoting medical quality [4]; thus, the performance analysis of medical service quality has important significance to provide indicators for improving the operations of medical institutions. However, due to limited and compartmentalized medical resources, it is impossible for managers to fully improve medical service quality [5].

Performance measurement of medical institutions’ service quality plays a very important role in hospital management and attracts more and more attention from relevant senior managers of medical institutions. As medical institutions provide diagnosis, treatments, and rehabilitation services, it is essential to improve the service quality and performance of these medical institutions. Therefore, appropriate measurement methods that can evaluate the medical service quality of medical institutions and determine good medical management methods to provide high-quality medical care services are medical-related problems that medical institutions must solve immediately [6].

Efficient and safe performance measurements of medical service quality can improve the quality of medical services, reduce the cost of related medical institutions, optimize related medical service processes, and achieve reasonable resource allocation, which would result in improved medical management and operational performance [7]. Due to increasing demands for medical services, the need for government supervision of medical health institutions has increased, and various problems, such as how to provide patients with high-quality medical services and improve patient satisfaction, have attracted the attention of relevant senior management levels of medical institutions [8].

The medical industry is a very competitive and professional field for private medical institutions, and the performance of medical service quality is relatively important for improving finance and patient satisfaction. The measurement of medical service quality performance provides medical institution managers with information about their medical performance; thus, the collection and analysis of problems must be improved [9]. In medical institutions, service performance can be affected by many indicators; thus, it is unrealistic to monitor and manage them all due to the limited medical resources. Improving the performance of medical services is related to the benefits of many medical departments; thus, a large number of experts with different backgrounds must participate in the evaluation of medical service performance measurements [10].

At present, as the performance measurement of medical service usually includes many indicators, medical service quality cannot be completely improved based only on the limited resources of medical institutions. Therefore, a limited number of key performance indicators for medical institutions must be determined to measure and monitor the performance of medical institutions’ service quality and provide medical institutions with safe and efficient working conditions in order to achieve patient/staff satisfaction through proper and efficient management [10]. More and more attention has been paid to literature regarding the evaluation and measurement of the improvement processes of medical service quality. Achieving high-quality medical service requires reliable management information, accurate problem identification, and rigorous analysis of relevant data, as well as the ability to measure and re-measure the performance of constructed medical service quality [11]. Therefore, this study attempts to divide the service performance measurements of medical institutions into meaningful indicators and investigate the relative importance among them. This study will also analyze the correlations among necessary medical service performance indicators to determine the primary indicator affecting medical service performance. This study mainly focuses on the key measurements of medical institutions’ service performance and explored which decisive ones could strengthen and improve the competitiveness of medical institutions’ service quality. Medical institutions refer to hospitals and clinics in this study. This study analyzes the standards of medical institutions’ service performance according to the dimensions of the balanced scorecard (BSC). The BSC has been regarded as an effective tool for medical and health management and operation [12,13,14]. Therefore, this study introduced BSC into medical institutions to improve their operational performance. Although the BSC has been applied to medical institutions in the literature [12,13,14], much remains to be learned about its systematic hierarchical structure of medical service performance in medical institutions. To fill the research gap, this study attempts to build a suitable BSC evaluation indicator structure determined based on the analytic hierarchy process (AHP) and expert questionnaires were made. As there might be correlations among the various relatively important key indicators, this study will also quantify the causality of service performance measurements of medical institutions in Taiwan through decision making and trial evaluation laboratory (DEMATEL).

## 2. Materials and Methods

### 2.1. Developing Service Performance Measurements

The BSC is an organizational performance management system introduced by Kaplan and Norton in 1992 [15,16]. In order to solve the problem that management only focused on financial performance in the short term to address the poor design of the performance measurement system, Kaplan and Norton cited the BSC performance measurement system, which integrated corporate financial and non-financial operation indicators and performance management systems [15,16]. Traditionally, only the return on investment (ROI) and financial accounting of the payback period were considered, which limited the performance management of organizational growth, adaptability, and competitiveness of a company. Therefore, BSC gained a dominant position to avoid institutional managers only emphasizing the long-term benefits brought by short-term financial performance indicators, such as new product development, process improvements, human resource developments, information technology, and customer and market developments, while opportunities for future investment growth were limited [17]. Among the four dimensions of BSC (finance, customer, internal processes, and learning and growth) [9], the former is a traditional financial performance dimension, while the latter three are non-financial performance measurement indicators, namely, the customer dimension, internal process dimension, and learning and growth dimension. BSC evaluates measures and guides enterprises’ specific functions and field items according to the specific factors of the organization or industry. The relative weights of the BSC performance dimensions can be applied in many fields, including medical institutions [2,4,8]. Therefore, the four dimensions of BSC, finance, customer, internal process, and learning and growth, are adopted in this study. In this study, literature collection is based on balanced scorecards as the management basis of medical institutions to establish operation and management performance indicators. Meanwhile, treating achieving the strategy and vision of medical institutions as the core basis, this study searched for related management performance indicators of medical institutions for discussion and research. According to relevant literature and the suggestions of experts and scholars, the evaluation dimensions and indicators of service performance measurements for medical institutions are summarized in Table 1. References from relevant experts or researchers were collected to support the content of relevant BSC service indicators, which are unanimously agreed by most national medical institutions to influence the management of service indicators [12,13,14,18].

#### 2.1.1. Financial Dimension

The financial dimension of medical institutions refers to the economic performance indicators related to the profitability of each hospital unit, and the following measurement factors were formulated: cost structure of improving equipment [17,19,20], medical incomes of institutions [20,21], return on assets (ROA) of the institution [8,17,20,21], cash flow of the institution [8,17,21], and economic added value [17,22]. Medical institutions with good financial status can purchase advanced medical instruments to improve the level of medical hardware equipment. In a large-scale medical institution, all units have common needs for medical aids, such as medical gloves. In this way, medical consumables can be purchased collectively by all units through vertical integration with the scale of medical institutions, namely the economic value added to the medical institution scale to reduce procurement costs [13,18].

#### 2.1.2. Customer Dimension

Patients are the source of income for medical institutions; thus, the goal of medical institutions is to meet patients’ demands for professional medical services. After the expected goals of medical institutions were determined, customers and medical field markets were compartmentalized, and the following measurement indicators were formulated: classification of medical profession according to customers (VIP system) [8,23], complete and comfortable equipment [23], continuity in patient-to-hospital treatments [8,17], competitiveness of the medical profession [8,20], complete medical services [4,8,20,21], and promotion through patient’s word of mouth [8,17,22]. After the patient enters the medical institution, the latter provides the patient with relevant medical examination and treatment items, according to which applications can be sent to the government health insurance institution for medical income. Medical institutions can provide patients with more complete and comfortable equipment based on regular updates and maintenance of equipment, and completion of the units and equipment of medical institutions can provide patients with more complete medical services. For example, a car accident patient entering a small medical institution may fail to be diagnosed with bone fractures due to the lack of X-ray equipment. Therefore, it is important for medical institutions to have complete and comfortable facilities and to provide patients with complete medical care.

#### 2.1.3. Internal Process Dimension

The operational processes of medical institutions are intended to meet patients’ demands for medical services, achieve the maximum financial target of the organizations, and establish a complete value chain of internal medical business processes in hospitals that can meet current and future demands. Therefore, the following measurement indicators were formulated: complete policy and promotion [21,22,23], quality of system software [19,23], real control of planning and implementation [19,23], optimization of unit operational processes [19,20], innovation and social responsibility [17,20,22], and continuous follow-up service for patients discharged from hospital [17,22]. If the managers of medical institutions are not medical professional managers, or relevant complete regulations and staff promotion standards for medical institutions are not formulated, or relevant regulations in accordance with the standards are not implemented, outstanding medical staff in medical institutions will quit due to incomplete policies and promotion, which will lead to low-quality medical services and management crisis in medical institutions.

#### 2.1.4. Learning and Growth Dimension

The development and motivation of the internal professional human resources of organizations are emphasized, and long-term growth and improvement of the medical profession can be achieved by staff, systems, and organizational programs. The growth of employees’ medical profession is an intangible asset for medical institutions and is helpful for the sustainable development of medical institutions. Therefore, the following measurement indicators were formulated: employee growth (education and training) [17,22,23], the organization and development of internal staff [17,20], complete salary, remuneration, policies [17,19], competent managers [20], and the introduction of new technologies [17,22]. Employee growth refers to the improvement of the medical professional level of employees through professional medical training. Internal employee organization and development mean that the medical institution has a complete standard of employee promotion so that employees can achieve smooth promotion through this organization.

### 2.2. Combining AHP with DEMATEL

AHP was developed by Saaty [23] in 1971 at the University of Pittsburgh; U.S. AHP is a nonlinear framework that can implement the thinking modes of deduction and induction and simultaneously consider many factors without using syllogisms. In order to conduct comparisons of pairs of factors at each layer, the AHP evaluation scale can be divided into nine scales: equal importance, moderate importance, strong importance, very strong importance, extreme importance, and four other scales. The evaluation values of the five levels of the nominal scales are 1, 3, 5, 7, and 9, while those of the other four levels fall between those five evaluation values, namely 2, 4, 6, and 8 [24].

AHP is a multiple attribute decision making tool, which allows the consideration of financial and non-financial quantitative and qualitative measures, provides a reasonable basis for managers to make decisions, and integrates different measurement standards to obtain a weighted ranking [24]. By using systematic technology to compare and evaluate the relative importance between two or more attributes in pairs, all problems can be treated as entities for study, and the interactive relations between the hierarchical components can be simultaneously examined. In the layered system model, all factors can be arranged systematically according to their priority weight, and their contributions to risk can be explained; thus, it is a powerful tool to cope with qualitative and quantitative multi-standard factors in the decision-making process. The steps include using hierarchy to classify decision-making problems into sub-problems that are easy to understand and evaluate in order to determine the weights of elements at each decision-making layer; then, determining the general weight order of the decision-making plan and building the hierarchical structure according to priority weight ranking in order to confirm the relative importance of all elements at each layer of the hierarchical structure. To ensure consistent judgments, the results should undergo consistency verification at the end. The degree of consistency between pairwise comparisons can be calculated by the consistency ratio. If the consistency ratio exceeds the limitation, the pairwise comparisons should be reviewed and corrected. When judging a hierarchical structure, Saaty [23] suggested that the CR should be less than or equal to 0.1 in order to meet consistency requirements. This system can be applied to address extensive problem fields from simple to complex capital-intensive decision making. As the success of this theory is based on its simplicity and steadiness, it is used in group interactions and decision-making systems, as well as to solve complex decision-making problems in different fields, such as planning, resources assessment, performance measurement, resource allocation, selecting the best strategy, and setting priorities [24,25].

AHP can be decomposed into dimensions and factors to systematize original complex problems and assumes that all dimensions or sub-standards are independent conditions, meaning that AHP only concerns the direct relations or relative weights between dimensions and sub-standards but cannot analyze the causality between them. Therefore, this paper uses DEMATEL to analyze whether there is causality between standards. In addition to analyzing the causality between standards, DEMATEL could make up for AHP’s limitation by allowing a better understanding of the complex interactions between standards.

The DEMATEL technique was initially developed by the Geneva Research Centres of the Battelle Memorial Institute. Developed by Gabus and Fontela [26], DEMATEL is an effective technique to assess measurement indicators and is applied in many fields. DEMATEL explains the correlation between factors and visualizes those factors into causal groups [27], which can establish an incidence matrix of influencing factors. In addition, the groups can be further divided into causal groups and influencing groups according to their influence on specific systems. In terms of the performance measurement and improvement of medical institutions, some indicators examine how the quantitative performances of medical institutions act upon each other [27]. DEMATEL is a simple and effective method and has important value in inspecting the interdependence of healthcare-related indicators for performance improvement in medical institutions [5,10,28]. The main strengths of DEMATEL are its highlighted presentation and its ability to identify the causality between factors. As AHP cannot explain causality [24], the DEMATEL technique is extensively used by researchers as it can determine the overall influencing degree of factors when analyzing relative factors, meaning it can classify the factors into causal groups and establish causality. Hence, this study used AHP to measure the priority of medical service performance indicators of medical institutions and used DEMATEL to assess the causality among those medical service performance indicators.

### 2.3. Participants and Data Collection

The collection of investigation information was conducted at the senior management level of the medical institutions, including the deans (the person in charge), deputy deans, and directors of medical institutions, through a questionnaire survey. As it was difficult to obtain a list of all senior operation managers of the medical institutions, a sample questionnaire survey was conducted among senior managers with practical management experience in district hospitals and clinics, as well as other medical institutions. As this study adopted AHP and DEMATEL, it was not necessary to obtain a large number of questionnaires from experts during the process of opinion collection [25,28]. Therefore, 52 experts at the director level or above with management experiences in medical institutions were invited to participate in the questionnaire survey. The researchers contacted them through emails and phone calls and sent them a questionnaire and a self-addressed envelope by post in order to collect the practical operations and management experiences of the sampled district hospitals and clinics. After collecting the questionnaires, the sampled experts were contacted through emails or phone calls to confirm their questionnaire answers [23]. If the questionnaire failed to meet the consistency ratio of being equal to or less than 0.1, or the interviewee failed to complete the questionnaire, the questionnaires were deemed invalid.

As one of the authors is a medical administrator, he has participated in the operation and management of seven hospitals and two clinics in Taiwan, which are mainly located in the southern and central regions of Taiwan. Therefore, the questionnaire was filled out by the administrators of medical institutions he is familiar with, so as to fully understand which operation and management indicators of medical institutions will affect the operation performance of medical institutions and the cause–effect relationship among relevant medical service indicators. Among the collected 52 questionnaires, 43 were valid questionnaires that conformed to the consistency check. The respondents of these 43 valid questionnaires included 27 males (62.8%) and 16 females (37.2%). Nineteen (44.2%) of them were from the public sector, and twenty-four (55.8%) were from the private sector. Twenty-one (47.6%) of them were deans, seven (16.3%) were deputy deans, and fifteen were directors responsible for management (34.9%). In terms of qualifications, there were 4 persons (9.3%) with 10 to 14 years of management experience, 6 persons (14.0%) with 15 to 19 years of management experience, 4 persons (9.3%) with 20 to 24 years of management experience, 16 persons (37.2%) with 25 to 29 years of management experience, and 13 persons (30.2%) with more than 30 years of management experience. All of those 43 persons passed the consistency check, and their questionnaires were suitable for AHP analysis.

## 3. Results

### 3.1. AHP Results

The analysis results of the weight values of all dimensions and service performance measurements are shown in Table 2. From the perspective of the assessment dimension, management experts in medical institutions believed that the customer dimension (0.4235) is the most important one, the second is the internal process dimension (0.2192), and the third is the learning and growth dimension (0.1877), followed by the financial dimension (0.1695).

In the financial dimension, the most important critical service performance indicator is the “medical incomes of institutions”. In terms of customer dimension, “complete and comfortable equipment” is the most important critical service performance indicator. In the internal process dimension, “complete policy and promotion” is the most important critical service performance indicator. In terms of the learning and growth dimension, “complete salary, remuneration, and policies” is the most important critical service performance indicator.

Several researchers have suggested that each company or industry could have two to six factors that can decide its success in operations [24,25]. Hence, this study concluded some key service performance indicators that may make medical institutions succeed in operations. According to the results of this study, as shown in Table 2, there was a small difference between the values of comprehensive weights ranked No. 6 (0.0563) and No. 7 (0.0539), while there was a large difference between the values of comprehensive weights ranked No. 7 (0.0539) and No. 8 (0.0472). Thus, the first seven critical service performance indicators for medical institutions are “complete and comfortable equipment”, “competitiveness of the medical profession”, “continuity of patient-to-hospital treatment”, “classification of medical profession according to customers (VIP system)”, “complete medical service”, “complete salary, remuneration, and policies”, and “medical incomes of institutions”.

### 3.2. DEMATEL Results

D + R is the correlation degree. The higher the correlation degree of the selection service criteria, the more important the service criteria are in the operation and management of medical institutions, and the medical institutions are also more willing to improve these service criteria. The positive value in the relation (D − R) indicates that the service criterion is the cause criterion (cause), and the administration level of medical institutions has great flexibility to adjust and improve such service criterion. The service criterion in the first quadrant (characteristic factor with core influence) is a core influential factor among many service criteria and should be listed as the priority factor of administrators. The first priority of management resource input is the service criterion in this quadrant, and correlation degree (D + R) and relation (D − R) are high key factors in service indicators. The indicator in the second quadrant (the indicator with driving factor characteristics) is the driving influence indicator among many indicators. The characteristic of this quadrant is low correlation degree (D + R) and high relation (D − R). The third quadrant (the indicator with independent factor characteristics) is where the risk indicator with low correlation degree (D + R) and relation (D − R) will fall into. The index in this quadrant belongs to the outcome factor and has low interaction with other factors. The factors in the fourth quadrant (the indicators with the characteristics of the affected factor) are the affected factors among many other factors, and the indicators in this quadrant are the key indicators for the high correlation degree (D + R) and the low relation (D − R).

**Figure 1:**
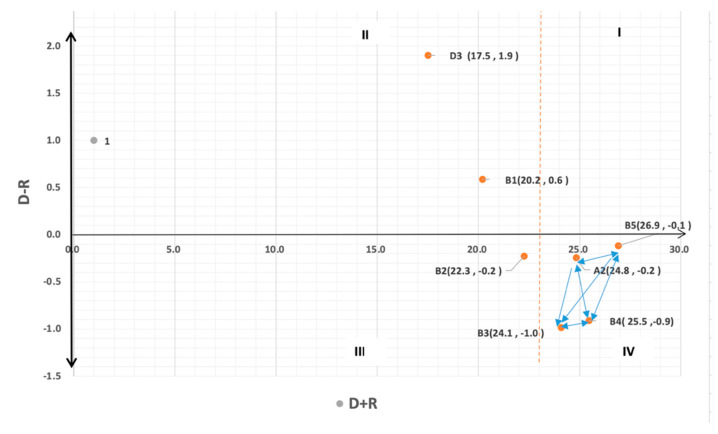
Cause–effect diagram of seven service performance indicators.

The cause–effect diagram of the seven key service performance indicators, as shown in Figure 1, illustrates the complex causality among them. The values of four service performance indicators, including medical incomes of institutions (A2), continuity of patient-to-hospital treatment (B3), competitiveness of the medical profession (B4), and complete medical service (B5), were located in the right of D + R (correlation degree) of the cause–effect diagram. Furthermore, the calculated D+R values of these four dimensions were all greater than the average value of 23.0. Hence, it can be learnt that compared with other service performance indicators, these four service performance indicators had a higher degree of correlation. Furthermore, because the D − R values of classification of the medical profession, according to customers (B1) and complete salary and remuneration policy, were greater than 0, this indicates that they belonged to cause standards, namely the “cause” in causality. However, because the D − R values of complete equipment and comfortability (B2), medical incomes of institutions (A2), continuity of patient-to-hospital treatment (B3), the competitiveness of the medical profession (B4), and complete medical service (B5) were less than 0, they belonged to the influenced performance indicators, namely the “effect” in causality.

## 4. Discussion

### 4.1. Priority of Service Performance Measurements

According to the AHP results, this study concludes seven critical service performance indicators for medical institutions. The following discussion addresses these indicators.

#### 4.1.1. Complete and Comfortable Equipment (B2)

Patients emphasize the importance of functional equipment in medical institutions, including physical desks with attending service staff in the temporary consultation period to provide guidance for patients and pacify them. In this way, patients can have a comfortable feeling and feel at home. Complete equipment and cleanliness are also necessary for medical institutions [29]. In addition, good equipment is an important factor in the operation and management of medical institutions [30]. The key features and cornerstone of a high-quality health care system in medical institutions are adequate basic health care facilities with bright lighting equipment and complete electric equipment.

Nowadays, medical equipment in medical institutions requires constant updating as the service life of equipment is shortened due to the rapid advances in technology. Medical equipment is often expensive and may cost tens of millions or more; thus, the considerations of investment costs and updating equipment is a problem for hospital operators in terms of achieving sustainable operations. Currently, the development of clinical specialties in medical institutions depends on the development of medical instruments to a large extent. The modernization of equipment in medical institutions is an important symbol of medical modernization; thus, the construction and management of medical equipment have been important parts of the management of modern medical institutions. Maintaining the good operational status of a medical equipment management system can improve the operation and management value of medical institutions. Social benefits, economic benefits, and technical feedback of hospitals are the goals of medical institution management; thus, the improvements in medical technology cannot be separated from two factors, namely talents and equipment. Advanced medical equipment is the basis for improving medical technology, and the introduction of advanced medical equipment is an indispensable condition for improving medical technology.

#### 4.1.2. Competitiveness of the Medical Profession (B4)

From the perspective of patients, ethical decision making involves medical professional doctors and many essential features, such as mercy, trust, and integrity. The maximum transparency of informed consent (voluntarily) from patients is the best model to establish a physician–patient relationship. Allowing patients to be familiar with and understand the diagnosis made by physicians, as well as relevant medical treatment plans, including medical risks and prognosis, is the most basic service performance indicator to gain trust in the medical profession. Medical institutions should focus on developing clinical technique management skills to improve their medical system and organization. Moreover, medical colleges and medical professional institutions can develop professional skills related to medical treatment through relevant medical education [31]. When measuring the quality of medical services, patients consider their functional quality and technology quality. Technology quality includes the professional services provided by relevant staff in medical institutions, adequate diagnostic testing, and appropriate treatment [28]. Patients often require both medical resources and multi-professional medical treatments; thus, high-quality medical treatment requires the integration of various medical-treatment-related resources to improve the quality of life and physical condition of patients. As service quality is sometimes lower than patients’ expectations, improving the quality of medical care service in medical institutions can provide more effective services to improve the health condition of people in the community. In order to narrow the gap in medical service quality, as perceived by patients, employees of medical institutions should be appropriately trained to professionally communicate with patients when they are receiving medical services [32].

The biggest problem with medical treatments in medical institutions in Taiwan is not the National Health Insurance system but the system that provides the actual professional medical services. In fact, improving medical expertise will improve the quality of medical treatment, better control the medical expenses of patients, and improve the service quality of medical staff, thus, increasing patients’ satisfaction with medical services. The medical industry is a specialized industry, and quality medical treatment is the core of the operations and management of medical institutions, as well as the foundation of the competitiveness of medical institutions. Improving medical expertise in medical institutions is the best way to develop quality and efficiency in the profession. In order to create more benefits for medical institutions, effective cost measures should be used to provide high quality professional medical services. The aims and goals of hospitals are to treat a wide range of diseases and sicknesses, as well as to save patients, while the goal of the patient is to have their illness treated; therefore, the first factor for patients to consider when choosing medical institutions is the medical expertise of the institution, followed by relevant services, environment, and other factors. When a patient’s needs are met, they would be willing to recommend the medical institution through word of mouth. Among all factors that affect patients’ decision to choose a medical institution, medical expertise is always the service performance indicator that patients pay close attention to; hence, quality medical expertise is the core value and goal of medical institutions.

#### 4.1.3. Continuity of Patient-to-Hospital Treatment (B3)

Being confronted with the crisis of professional human resources, the solutions for reducing the death rate and the loss rate of follow-ups will be a win–win situation for medical institutions to gain patients’ loyalty. In this study, critical service measurements to improve the retention rate of patients have been proposed, including a simple and standardized medical monitoring system to confirm patients’ real conditions, a guarantee of continuous supply of drugs, the reduction in patients’ indirect costs (e.g., costs related to transportation generated from the journey to and from medical institutions), and the reinforced connection between medical and health services, as well as internal communities (community medical treatments) [33].

The retention of patients in medical institutions is a complex problem, which depends on all relevant interrelated factors. The design of the health care system in medical institutions is based on the measuring standard of the quality of the retention rate of patients. Medical institutions should try to understand and assess the reasons why patients stop treatments in their institutions [34].

If medical institutions want to create a professional medical care system with a high level of quality, they must be patient oriented. They should design all medical professions and services for patients based on their health conditions as what enterprises do. In the past, enterprises were organized by production and marketing; however, several years ago, they changed to a divisional structure, becoming customer oriented and organizing technologies and services required by customers. Only if the medical industry is patient-oriented can it bring more value to patients in the whole course of medical treatments, including monitoring, precaution, diagnoses, treatments, and ongoing management of patients’ conditions. As for the question of how to improve the patient-oriented professional medical service system, a triple-win value for patients, medical personnel, and medical institutions should be created. Patients’ participation is important for their health outcomes. These “products” under professional medical care must be created by medical personnel and patients together.

#### 4.1.4. Classification of Medical Profession according to Customers (VIP System) (B1)

As consumers, patients have rights and obligations. They have the right to upgrade their medical treatment level according to their economic capability, so as to meet their desire to gain comfortability at the time of receiving medical services. Primary factors driving them to choose medical institutions of high quality include doctors’ care, detailed consultations, participation in treatment-related decisions, long-tested curative effect (for patients with chronic diseases), active and quick responses of medical personnel to assist in a medical procedure, comprehensive medical equipment, and five-star medical facilities. What they need is to bear the expensive costs of medical care services. However, all interviewees (patients) have paid or were willing to pay relevant medical costs by purchasing insurance [35]. Patients choose to upgrade their medical treatment level. They do not need to pay extra medical costs if they choose not to upgrade their medical treatment level. Factors that influence the tendency to upgrade to the VIP medical treatment level include perception, knowledge, income, and family support [36].

The VIP system of professional medical services is medical services taking medical care, sickness and health, rehabilitation, and recuperation as the themes. At present, the medical service industry with international competitiveness should not only meet the needs of domestic citizens for professional medical services but also attract foreigners who have the need for professional medical services. In addition to improving medical institutions’ operation and management efficiency, it can also increase the economic value of domestic tourism and other service industries, as well as developing international medical tourism. Currently, international medical tourism is gaining popularity.

#### 4.1.5. Complete Medical Service (B5)

The development of medical institutions resulted in competition for health care facilities. Patients’ needs for high quality medical services increase every day. Higher educational levels and community economy require medical institutions to establish a complete plan for their medical service systems to meet patients’ demands and desires for high-quality medical services (customer oriented). Given the expensive operating costs of hospitals, medical institutions should strive for survival and development. The service quality expectation they should aim for is to increase their income from patients as patients are the direct source of income or indirect source of income gained from health insurance for hospitals. Without patients, medical institutions cannot survive and develop. In order to allow more patients to receive treatments in their institutions, hospitals must provide comprehensive medical services for patients and increase patients’ satisfaction with high-quality medical services [37]. Patients who receive medical services will form corresponding satisfaction with the quality of medical services according to their opinions on the quality of medical services. The research result of this study revealed that there was a relationship existing between the quality of medical services and patients’ loyalty. Therefore, the improvement of the overall professional competence of medical services in medical institutions played an important role in enhancing patients’ perception of medical services. The quality of medical services had a direct influence on patients’ loyalty. This finding is consistent with previous studies. Medical services of high quality can meet patients’ various demands so as to raise their willingness to go to the hospitals for treatments [38]. Providing medical services of good quality to gain patients’ loyalty has become a key factor for medical institutions to survive and develop. When patients need hospitalization, they will choose the hospital they trust, and such trust comes from their loyalty to hospitals and satisfaction with medical services.

#### 4.1.6. Complete Salary, Remuneration, and Policy (D3)

Salary and remuneration play important roles in improving employees’ welfare and enhancing employees’ performance and job satisfaction. However, there are still some employees that are not satisfied with their salary and remuneration. Nonetheless, salary and remuneration are very important in the human resources management of medical institutions. Furthermore, salary and remuneration are the tools for medical institutions or enterprises to bind themselves with their employees and the factors to drive employees to work hard and increase their satisfaction. In addition, salary and remuneration have a very important impact on organization operations. The use of human resources and the salary and remuneration policy of medical institutions with high efficiency allows medical institutions to achieve stable and growing performance and income. A successful salary and remuneration policy can automatically enhance employees’ work performance and create harmonious cooperation, which satisfies all parties [39]. Therefore, the management of medical institutions must establish sound salary and remuneration policies and provide employees with allowances to stimulate employees’ working enthusiasm. Meanwhile, medical institutions should strengthen the communication between the management and employees through clear SOP, especially in terms of using the salary and remuneration policy as a tool to stimulate employees’ working enthusiasm.

#### 4.1.7. Medical Incomes of Institutions (A2)

From the perspective of the impacts on the market structure of medical institutions, will the price and income shock lead to the opening or closing of specialized departments or even the hospital by medical institutions? If specialized departments can provide nursing and care with higher quality, it plays an important role in patients’ welfare, even though it has no influence on the total number of hospitalizations. Changes in the medical incomes of institutions will influence the execution of relevant treatment departments of medical institutions, and the effect of medical incomes of institutions will influence practical treatment decisions of medical institutions. Hence, the medical incomes of institutions can be regarded as a significant factor in the operation of institutions [40].

Should medical institutions make profits? In terms of medical institutions, for the goal of saving lives and improving the health of more patients, medical institutions need to continuously update medical equipment, enhance the level of the medical profession and use new medical expertise to improve the level of patient care. Both the cultivation of medical personnel and the procurement of new instruments are necessary investments. Therefore, medical institutions must have some surplus to do these things.

### 4.2. Causality of Service Performance Measurements

According to the DEMATEL results, this study also investigates the cause–effect relationships among the seven key service performance indicators. The operation and management of medical institutions present a great difficulty. If the operation direction and development strategy of managers in medical institutions are correct, medical institutions will gain good operation performance and strong competitiveness. In Taiwan, under drastic changes in the medical environment and the Global Budget System of National Health Insurance, the competition in the medical market is fierce. Both the top managers who are responsible for the success or failure of the operation and management of medical institutions and the persons in charge of various departments in medical institutions must have an in-depth level of study and understanding of the essential concepts of the operation and management of medical institutions. They should also apply the operation and management theories to the practical operations of medical institutions. It is an important decision to determine the method to stand out among many competitors and maintain their sustainable competitiveness for medical institutions. Among the medical service performance indicators of medical institutions, which should be emphasized to achieve effective improvement and enhance the competitiveness of medical institutions? According to the cause–effect diagram of seven service performance indicators, D + R is the correlation degree. A higher correlation degree of selected service performance indicators indicates a higher degree of importance in the operation and management of medical institutions. Medical institutions also have a higher degree of willingness to improve on those service performance indicators. Hence, from the perspective of correlation degree (D + R), among the seven service performance indicators, medical institutions are more likely to improve the following service performance indicators, which have a higher correlation degree: Medical incomes of institutions (A2), continuity of patient-to-hospital treatment (B3), the competitiveness of the medical profession (B4), and complete medical service (B5). In addition, if the relation is positive (D − R), this indicates that the service performance indicator is a cause standard (cause), and medical institutions can make more flexible adjustments and improvements for such service performance indicators. Hence, from the perspective of relation (D − R), two service performance indicators of the seven service performance indicators belonged to cause standards. They are the classification of the medical profession according to customers (B1) and a complete salary and remuneration policy. The correlation degree (D + R) represents the importance degree of service performance indicators in the operation and management of medical institutions. Complete medical service (B5) is the most important service performance indicator, while a complete salary and remuneration policy are less important.

From the directions of arrows in Figure 1, it is noted that complete medical service (B5) can influence the competitiveness of the medical profession (B4), medical incomes of institutions (A2), and the continuity of patient-to-hospital treatment (B3). This is the service performance indicator with the highest correlation degree. Therefore, the most critical service performance indicator that requires managers of medical institutions to improve and address is complete medical service (B5) because as long as the medical services of medical institutions are improved, the continuity of patient-to-hospital treatment (B3), competitiveness of the medical profession (B4), and medical incomes of institutions (A2) will be enhanced. Thus, it can be concluded that complete medical service (B5) is the most critical service performance indicator to be addressed and improved by managers of medical institutions.

### 4.3. Limitations and Suggestions

A questionnaire was adopted in this study. The weight ranking and influence degree of the dimensions and service criteria were based on the opinions of the persons in charge of the operation and management of medical institutions. The present study took medical institutions in Taiwan as research objects. One limitation of this study is the restricted external validity as the sample frame is restricted to medical institutions in Taiwan. Making generalizations about the priority and causality of service performance indicators in other countries based on the present research findings may not be appropriate without further research. Future research can extend and replicate the current study in more countries. Furthermore, this study proposed 22 indicators to build the service performance measurements of medical institutions. We did not take the medical institution types into account in the current study. Medical institutions can be categorized into different types, e.g., public and private sectors; large, medium, and small hospitals. Future research can extend and replicate the current study to investigate the differences in the priority and causality of service performance indicators among different types of medical institutions.

## 5. Conclusions

This study worked out 4 dimensions and 22 service performance indicators by reviewing literature related to service performance indicators of medical institutions. AHP and DEMATEL questionnaires were designed according to the summarized dimensions and service performance indicators. This study judged and assessed relative weights and causality with the help of the persons in charge of operation and management in medical institutions. According to the results of AHP, the customer dimension was the most important dimension to study the medical service performance indicators of medical institutions. The seven critical factors influencing the medical service performance indicators of medical institutions were “complete and comfort equipment”, “competitiveness of the medical profession”, “continuity of patient-to-hospital treatment”, “classification of medical profession according to customers (VIP system)”, “complete medical service”, “a complete salary and remuneration policy”, and “medical incomes of institutions”. From these study results, it could be found that the customer dimension was the most important weight among the four dimensions. This is consistent with the medical institutions’ patient-oriented aim in practice. Therefore, to have competitiveness, medical institutions must be patient-oriented and possess advanced medical equipment, complete medical service, and medical personnel with competitiveness in the medical profession. However, they must also have a complete salary and remuneration policy to retain good medical personnel for providing patients with professional medical services. In this way, patients will feel at home and would like to continue receiving treatments in their hospitals. As a result, the overall income of medical institutions will be increased, and the medical service quality of medical institutions will be comprehensively enhanced. This is a win–win outcome. Based on the results of DEMATEL and according to the cause–effect diagram of seven service performance indicators, it was found from the results that complete medical service influenced the competitiveness of the medical profession, medical incomes of institutions, and continuity of patient-to-hospital treatment, and it is also the service performance indicator with the highest correlation degree. Hence, it can be concluded that complete medical service is the most critical service performance indicator to be addressed and improved by managers of medical institutions. This study contributes to the literature by integrating the AHP and DEMATEL approach and the balanced scorecard concept to build a service performance measurement system for medical institutions.

## Figures and Tables

**Table 1:** Service performance measurements of medical institutions.

Dimension	Dimension Description	Indicator	Indicator Description
Financial dimension (A)	The financial perspective of medical institutions includes economic performance indicators related to the profitability of hospital units.	(A1) Cost structure of improving equipment	Introduce high-tech medical technology, increase equipment expenditures, and improve the medical level of units.
		(A2) Medical income of the institution	Increase health insurance and the proportion of self-paid income.
		(A3) ROA of the institution	Manage the income, hospital bed occupancy rate, staff costs, average inpatient and outpatient income, and the expenditures of medical institutions to increase the asset income of medical institutions.
		(A4) Cash flow of the institution	Manage cash inflow and expenditures of medical institutions and the financial situations that have reached stable levels.
		(A5) Economic added value	Vertical integration of medical institutions, referral of departments, and collective procurement of medical appliances and drugs.
Customer dimension (B)	Patients are the source of income for medical institutions and meeting professional needs for the medical care of patients is the goal of medical institutions.	(B1) Classification of medical profession according to customers (VIP system)	Establish a medical VIP system and make medical differentiation to increase self-paid income.
		(B2) Complete and comfortable equipment	Construct a complete medical equipment environment to provide the best medical environment for patients.
		(B3) Continuity of patient-to-hospital treatment	Provide complete medical services so that patients can continue to go to the hospital for medical treatment in the future.
		(B4) Competitiveness of the medical profession	Establish medical specialties to enhance the competitiveness and differences of hospitals in the various fields of the medical professions.
		(B5) Complete medical services	Provide complete medical services to increase patients’ satisfaction with medical services.
		(B6) Introduction through patient’s word of mouth	Provide complete professional medical services so that previous patients can introduce new patients with medical needs to hospitals for medical related services.
Internal process dimension (C)	Handle the operational processes of medical institutions to meet the medical needs of patients, achieve the largest financial goal of the organization, and build a complete hospital internal medical business process value chain that can meet current and future needs.	(C1) Complete policy and promotion	Complete policies and promotion of medical institutions to increase the professional quality and centripetal force of staff and then increase their work performance.
		(C2) Software quality of the system	Improve software quality and updating of medical institutions to reduce labor operation costs.
		(C3) Real control of planning and implementation	The budget planning and implementation of each unit in medical institutions should be strictly controlled to reduce the error rate.
		(C4) Optimization of unit operation process	Unit processes must be optimized and feature easy communication in order to reduce time costs and give patients a warm feeling.
		(C5) Innovation and social responsibility	Take it from society, use it in society, take the patient as the center, put staff first, and implement the social responsibilities of medical institutions.
		(C6) Continuous follow-up service for patients discharged from hospital	Continuous follow-up care services for patients after they have completed their medical treatment in order to increase the warm feeling of medical institutions.
Learning and growth dimension (D)	The learning and growth of medical institutions focus on the development and encouragement of professional human resources in their organizations.	(D1) Employee growth (education and training)	Provide staff with complete medical professional training to improve the medical quality of staff.
		(D2) Organization and development of internal staff	Establish a complete promotion method for medical units, where staff can give full play to their medical professional ability.
		(D3) Complete salary, remuneration, and policy	Provide complete salary, compensation, and policies to improve staff satisfaction and centripetal force.
		(D4) Competent managers	Provide complete management promotion policies where managers can give full play to their strengths.
		(D5) Introduction of new technologies	Introduce advanced medical equipment and software to improve the medical level of medical institutions.

**Table 2:** AHP results of the service performance measurements.

Dimension	Weigh (Rank)	Indicators	Weight (Rank)	Integrated Weight (Rank)
	0.1695 (4)	(A1) Cost structure of improving equipment	0.2179 (2)	0.0370 (15)
		(A2) Medical income of the institution	0.3181 (1)	0.0539 (7)
		(A3) ROA of the institution	0.2145 (3)	0.0364 (16)
		(A4) Cash flow of the institution	0.1439 (4)	0.0244 (18)
		(A5) Added value	0.1056 (5)	0.0179 (22)
	0.4235 (1)	(B1) Classification of medical profession according to customers (VIP system)	0.1699 (4)	0.0719 (4)
		(B2) Complete and comfortable equipment	0.2071 (1)	0.0878 (1)
		(B3) Continuity of patient-to-hospital treatment	0.1736 (3)	0.0735 (3)
		(B4) Competitiveness of the medical profession	0.1899 (2)	0.0804 (2)
		(B5) Complete medical services	0.1506 (5)	0.0638 (5)
		(B6) Introduction through patient’s word of mouth	0.1089 (6)	0.0461 (9)
	0.2192 (2)	(C1) Complete policy and promotion	0.2159 (1)	0.0472 (8)
		(C2) Software quality of the system	0.1972 (3)	0.0432 (11)
		(C3) Real control of planning and implementation	0.2043 (2)	0.0448 (10)
		(C4) Optimization of unit operation process	0.1725 (4)	0.0378 (14)
		(C5) Innovation and social responsibility	0.1009 (5)	0.0241 (19)
		(C6) Continuous follow-up service for patients discharged from hospital	0.1004 (6)	0.0220 (20)
	0.1877 (3)	(D1) Employee growth (education and training)	0.2186 (2)	0.0411 (12)
		(D2) Organization and development of internal staff	0.2014 (3)	0.0378 (13)
		(D3) Complete salary, remuneration, and policy	0.2997 (1)	0.0563 (6)
		(D4) Competent managers	0.1684 (4)	0.0316 (17)
		(D5) Introduction of new technologies	0.1119 (5)	0.0210 (21)

## Data Availability

Not applicable.

## References

[1] Mainz J. (2003). Defining and classifying clinical indicators for quality improvement. Int. J. Qual. Health Care.

[2] Weir E., d’Entremont N., Stalker S., Kurji K., Robinson V. (2009). Applying the balanced scorecard to local public health performance measurement: Deliberations and decisions. BMC Public Health.

[3] Rogers A.E., Hwang W.T., Scott L.D., Aiken L.H., Dinges D.F. (2004). The working hours of hospital staff nurses and patient safety. Health Aff..

[4] El-Jardali F., Saleh S., Ataya N., Jamal D. (2011). Design, implementation and scaling up of the balanced scorecard for hospitals in Lebanon: Policy coherence and application lessons for low and middle income countries. Health Policy.

[5] Zhang L., Liu R., Jiang S., Luo G., Liu H.C. (2020). Identification of key performance indicators for hospital management using an extended hesitant linguistic DEMATEL approach. Healthcare.

[6] Soysa I.B., Jayamaha N.P., Grigg N.P. (2018). Developing a strategic performance scoring system for healthcare nonprofit organisations. Benchmarking.

[7] Christiansen T., Vrangbæk K. (2018). Hospital centralization and performance in Denmark—Ten years on. Health Policy.

[8] Behrouzi F., Ma’aram A. (2019). Identification and ranking of specific balanced scorecard performance measures for hospitals: A case study of private hospitals in the Klang Valley area, Malaysia. Int. J. Health Plan. Manag..

[9] Smith P., Mossialos E., Papanicholas I. (2008). Performance Measurement for Health System Improvement: Experiences, Challenges and Prospects.

[10] Jiang S., Shi H., Lin W., Liu H.C. (2019). A large group linguistic Z-DEMATEL approach for identifying key performance indicators in hospital performance management. Appl. Soft Comput..

[11] Abujudeh H.H., Kaewlai R., Asfaw B.A., Thrall J.H. (2010). Quality initiatives: Key performance indicators for measuring and improving radiology department performance. Radiographics.

[12] Vesty G., Brooks A. (2017). St George hospital: Flexible budgeting, volume variance, and balanced scorecard performance measurement. Issues Acc. Educ..

[13] Aidemark L.G. (2001). The meaning of balanced scorecards in the health care organization. Financ. Account. Manag..

[14] Northcott D., France N. (2005). The balanced scorecard in New Zealand health sector performance management: Dissemination to diffusion. Aust. Account. Rev..

[15] Kaplan R.S., Norton D.P. (1992). The Balanced Scorecard—Measures that drive performance. Harv. Bus. Rev..

[16] Kaplan R.S., Norton D.P. (1996). The Balanced Scorecard: Translating Strategy into Action.

[17] Yaghoobi T., Haddadi F. (2016). Organizational performance measurement by a framework integrating BSC and AHP. Int. J. Prod. Perform. Manag..

[18] Kober R., Northcott D. (2021). Testing cause-and-effect relationships within a balanced scorecard. Account. Financ..

[19] Perez C.A., Montequin V.R., Fernandez F.O., Balsera J.V. (2017). Integrating analytic hierarchy process (AHP) and balanced scorecard (BSC) framework for sustainable business in a software factory in the financial sector. Sustainability.

[20] Janeš A., Kadoić N., Begičević Ređep N. (2018). Differences in prioritization of the BSC’s strategic goals using AHP and ANP methods. J. Inf. Organ. Sci..

[21] Noori B. (2015). Prioritizing strategic business units in the face of innovation performance: Combining fuzzy AHP and BSC. Int. J. Bus. Manag..

[22] Lee A.H.I., Chen W.C., Chang C.J. (2008). A fuzzy AHP and BSC approach for evaluating performance of IT department in the manufacturing industry in Taiwan. Expert Syst. Appl..

[23] Saaty R.W. (1987). The analytic hierarchy process: What and how it is used. Math. Model..

[24] Ho W. (2008). Integrated analytic hierarchy process and its applications: A literature review. Eur. J. Oper. Res..

[25] Ho W., Ma X. (2018). The state-of-the-art integrations and applications of the analytic hierarchy process. Eur. J. Oper. Res..

[26] Gabus A., Fontela E. (1972). World Problems, an Invitation to Further Thought within the Framework of DEMATEL.

[27] Si S.L., You X.Y., Liu H.C., Zhang P. (2018). DEMATEL technique: A systematic review of the state of the art literature on methodologies and applications. Math. Probl. Eng..

[28] Glaize A., Duenas A., Di Martinelly C., Fagnot I. (2019). Healthcare decision-making applications using multicriteria decision analysis: A scoping review. J. Multi-Criteria Decis. Anal..

[29] Derriennic J., Barais M., Le Goff D., Fernandez G., Le Borne F., Le Reste J. (2021). Patient, carer and healthcare professional experiences of complex care quality in multidisciplinary primary healthcare centres: Qualitative study with face-to-face, in-depth interviews and focus groups in five French multidisciplinary primary healthcare centres. BMJ Open.

[30] Miller W.R. (2014). Patient-centered outcomes in older adults with epilepsy. Seizure.

[31] Walsh G., Hayes B., Freeney Y., McArdle S. (2019). Doctor, how can we help you? Qualitative interview study to identify key interventions to target burnout in hospital doctors. BMJ Open.

[32] Sharifi T., Hosseini S.E., Mohammadpour S., Javan-Noughabi J., Ebrahimipour H., Hooshmand E. (2021). Quality assessment of services provided by health centers in Mashhad, Iran: SERVQUAL versus HEALTHQUAL scales. BMC Health Serv. Res..

[33] Harries A.D., Zachariah R., Lawn S.D., Rosen S. (2010). Strategies to improve patient retention on antiretroviral therapy in Sub-Saharan Africa. Trop. Med. Int. Health.

[34] Brown H.J., Andreason H., Melling A.K., Imel Z.E., Simon G.E. (2015). Problems with using patient retention in the evaluation of mental health providers: Differences in type of dropout. Psychiatr. Serv..

[35] Bucatariu L., George B.P. (2017). Patient perception and choice factors related to international hospitals: A study in Ho Chi Minh City, Vietnam. J. Health Med. Inf..

[36] Purwatiningrum F. (2019). Analysis of factors affecting BPJS patients choosing a class of care to VIP room. J. Qual. Public Health.

[37] Putri A.D., Aulia D. (2020). Analysis quality of specialist doctor services and patient satisfaction at H. Abdul Manan Simatupang Hospital in Kisaran Regency. Int. Arch. Med. Sci. Public Health.

[38] Yu Y., Chen Z., Zhao Y., Wang Y., Zhang R., Zhou X. (2020). Medical service quality, psychological contract, and patient loyalty: An empirical study among patients in China. Medicine.

[39] Permana I., Bharoto H. (2021). Remuneration to improve employee performance at waled regional hospital, Cirebon regency. Int. J. Res. Bus. Soc. Sci..

[40] Bäuml M., Dette T., Pollmann M. (2022). Price and income effects of hospital reimbursements. J. Health Econ..

